# Lung cancer increases H_2_O_2_ concentration in the exhaled breath condensate, extent of mtDNA damage, and mtDNA copy number in buccal mucosa

**DOI:** 10.1016/j.heliyon.2020.e04303

**Published:** 2020-06-28

**Authors:** Natalya A. Kolbasina, Artem P. Gureev, Olga V. Serzhantova, Andrey A. Mikhailov, Ivan P. Moshurov, Anatoly A. Starkov, Vasily N. Popov

**Affiliations:** a Department of Genetics, Cytology and Bioengineering, Voronezh State University, Voronezh, Russia; b Voronezh Regional Clinical Oncological Dispensary, Voronezh, Russia; c Brain and Mind Research Institute, Weill Medical College of Cornell University, New York, NY, USA; d Voronezh State University of Engineering Technologies, Voronezh, Russia

**Keywords:** Biochemistry, Molecular biology, Cancer research, Oncology, Biological sciences, Lung cancer markers, Mitochondria, mtDNA damage, Hydrogen peroxide, Reactive oxygen species

## Abstract

We have shown that the H_2_O_2_ concentration in exhaled breath condensate (EBC) in lung cancer patients increases significantly compared to the EBC of healthy people and revealed the correlation between the H_2_O_2_ level in the EBC and amount of mtDNA damage in buccal mucosa cells. The H_2_O_2_ hyper-production may trigger mitochondrial biogenesis, thereby resulting in an increase in mtDNA copy number. However, we did not observe a significant difference in the studied parameters between smokers and non-smokers. Overall, our data suggest that H_2_O_2_ concentration in the EBC, the extent of mtDNA damage, and mtDNA copy number in buccal mucosa could be potential as an early diagnostic marker of lung cancer.

## 1 Introduction

Lung cancer is the most common type of cancer in the world, ranking first among the top five “killer cancers” (Benzaquen et al., 2019). The number of lung cancer cases is approximately equal to the death toll of this disease (Barta et al., 2019). In most cases, this is because more than 2/3 patients get specialized treatment at the stages of advanced tumor formation (Hoffman and Sanchez, 2017). Despite has been huge described scientific progress in oncology, cancer therapy is still hindered by the absence of reliable early diagnosis methods (Missel et al., 2015; Fitch, 2019; Behrend et al., 2003). Over the last decade, considerable progress has been achieved in cancer screening using different kinds of markers, such as genetic and epigenetic changes in nuclear DNA (Komerik et al., 2017) and quantitative changes in circulating RNAs (Di et al., 2019). However, these screening methods have certain disadvantages, such as invasiveness, high cost, and difficulties in the result interpretation related to the complexity of nuclear genome (Yu, 2011). All these limitations may be overcome with a new approach to the early lung cancer diagnosis using mtDNA, which might make cancer screening significantly easier, more sensitive, and accurate (Yu, 2011).

By activating redox signalling cascades, tumor cells cause a change in the cellular redox state, resulting in the production of the high level of reactive oxygen species (ROS), in particularly H_2_O_2_ (Ganeev et al., 2018). As a signal molecule in the bioenergetics pathway of prooncogenic signal transduction, H_2_O_2_ induces DNA damage, causing genome instability (Vilema-Enriquez et al., 2016). Due to their close proximity to the electron transfer chain (ETC), limited ability for repair, and absence of protective histones, mtDNA is about 50 times more sensitive to oxidative damage, as compared to the nuclear DNA (Chouteau et al., 2011). ROS cause oxidative damage to mtDNA, thereby affecting the efficiency of ETC functioning and outer mitochondrial membrane permeability (Ganta et al., 2017; Yadav and Chandra, 2014). The mtDNA damage promotes a release of pro-apoptotic agents that stimulate oncogenesis and tumor progression (Wallace, 2012; Larman et al., 2012).

DNA damage initiates tumorigenicity and maintains subsequent tumor development (Lennicke et al., 2015). Under pathological conditions, metabolic changes lead to the increase in the H_2_O_2_ concentration in exhaled breath. This can induce oxidative damage of mtDNA and affect mtDNA copy number in the buccal mucosa, rendering mtDNA as a potential early diagnostic marker of lung cancer and other diseases (Ganeev et al., 2018).

The aim of the work was to study the concentration of H_2_O_2_, as well as the number of lesions and the number of copies of mtDNA in healthy people and patients with lung cancer and to identify the relationship between these indicators.

## 2 Materials and methods

### 2.1 Human subjects

The participants of this study had been recruited among the patients observed for lung cancer at the Voronezh Regional Clinical Oncological Center (Voronezh, Russia) (experimental group) and healthy volunteers (control group). The control group was subdivided into 2 subgroups: smokers and non-smokers. Individuals in the experimental group did not suffer any respiratory system disease other than lung cancer and were not getting a treatment at the time of biological material collection. Patients with lung cancer had the following histological types: adenocarcinoma, solid tumor (uncharacterized histology), moderately differentiated squamous cell cancer and large cell cancer. The last two histological types of lung cancer were grouped into a "different" group. Lung adenocarcinoma is a tumor that develops from glandular tissue, which forms a peripherally located mass with сentral fibrosis and pleural contraction. A solid tumor is an aggressive type of epithelial cancer, which is a group of cells located in plates between layers of epithelial tissue and having a uncharacterized histology. For squamous cell lung cancer, there is a characteristic keratinization, as well as intercellular bridges. Tumor cells do not have a glandular structure or mucin production. In large cell lung cancer, large polygonal tumor cells form a solid leaf or nest (Table 1). The average age was similar in each group. The research protocol was approved by the Ethics Committee of the Voronezh State University and followed the key statements of the Helsinki Declaration. Informed written consents to the enrollment in the study were obtained from all participants that were informed about subsequent genetic tests conducted with their biological material.

**Table 1:** Detailed description of the experimental and control groups.

Parameter	Number of patients		
	Control (non-smokers) (n = 18)	Control (smokers) (n = 14)	Lung cancer patients (n = 14)
Age (mean ± SD)	54.28 ± 7.84	59.46 ± 7.39	62.61 ± 2.37
Sex: (male/female)	9/9	9/5	12/2

Lung cancer histology:			

Adenocarcinoma	N/A	N/A	8
Solid tumor (uncharacterized histology)	N/A	N/A	4
Other (large cell carcinoma, squamous cell carcinoma)	N/A	N/A	2

Stage:			

I	N/A	N/A	1
II	N/A	N/A	2
IIIA	N/A	N/A	5
IIIB	N/A	N/A	5
IV	N/A	N/A	1
Primary tumor (T_x_/T_1_/T_2_/T_3_/T_4_)	N/A	N/A	1/0/6/0/7
Regional lymph nodes (N_x_/N_0_/N_1_/N_2_/N_3_)	N/A	N/A	1/4/4/3/2
Distant metastases (M_x_M_0_M_1_)	N/A	N/A	1/12/1

### 2.2 Sampling of biological material

Cheek mucosa cells and EBC were collected from the lung cancer patients of the Voronezh Regional Clinical Oncology Center and healthy volunteers in April 2018 to February 2019 (Table 1).

Each participant was assigned an identifier that was then used for encoding collected samples for biochemical and molecular genetic studies. Cheek mucosa samples were collected by a medical professional by swabbing the inside of the cheek with a special FloqSwab brush and placing in a test tube containing DNA preserving solution (Helikon, Russia).

To obtain the EBC, the subjects were asked to breathe through a special mouthpiece for 5 min (Chen and Danao, 2013) while maintaining a certain breathing pattern (Gajdocsi et al., 2011). EBC was collected in a 15-mL tube, equipped with the inlet and outlet tubes and disposable mouthpiece (similar to that used in standard alcohol breathalyzers). EBC collection through the mouthpiece is more accurate, because when the samples are collected without the mouthpiece, the sample quality may be affected by the force of expiration, the distance from the person's lips to the device, the inclination angle, and the environmental conditions (the presence of alcohol vapor in the air, humidity, temperature, strength and direction of wind, etc.). To prevent sample contamination with saliva containing large amounts of the H_2_O_2_-decomposing catalase enzyme, a cotton swab was used. The collection tube was cooled with ice to precipitate the liquid from the exhaled air. The resulting EBC volume was over 100 μl. The liquid precipitate was placed in 50 ml tube and centrifuged at 500 g for 1 min. We have found earlier that storing EBC for more than 30 min resulted in H_2_O_2_ degradation; therefore, the H_2_O_2_ concentration was measured within 30 min after EBC collection.

### 2.3 H_2_O_2_ measurement

The H_2_O_2_ content in the EBC was measured by a fluorescence analysis (excitation wavelength, 555 nm; emission wavelength, 581 nm) using a Hitachi 7000 spectrofluorometer (Hitachi High-Tech, Japan) in three technical replicates. The measurement was conducted in an acrylic cuvette containing 1 ml of analysis buffer (20 mM HEPES-KOH and 1 mM EDTA), 10 mM Amplex®UltraRed (LifeTechnologies, USA), and 4 U/μL horseradish peroxidase (HRP) (Dikalov et al., 2007). Increment of H_2_O_2_ concentration was analyzed after addition of 15 μL of the sampled EBC. The calibration curve was plotted for 0.5, 1, 2, 4, 6, and 8 mM of freshly prepared H_2_O_2_.

### 2.4 mtDNA damage assay

DNA from cheek mucosa cells was isolated with a genome DNA extraction kit (Dia-M, Russia). The quality of the obtained preparation was assessed by electrophoresis in 2% agarose gel. DNA concentration was determined with a Qubit 2.0. fluorometer (Thermo Fisher Scientific, USA). The mtDNA damage was estimated by qPCR as previously described by (Gureev et al., 2017) using the 1903-bp DNA fragment that contained the *ND6*, *TRNE*, and *CYTB* genes.

The following primers was used for *ND6*, *TRNE*, *CYTB* genes:

F: 5′-AAACCCCATTAAACGCCTGG-3′
R: 5′-TCGGAGAATTGTGTAGGCGAAT-3′

To normalize the extent of mtDNA damage to the amount of mtDNA, a short 67-bp mtDNA sequence was amplified with the following primers (Lehle et al., 2014):

F: 5′-GGCCACAGCACTTAAACACA-3′
R: 5′-CCCTAACACCAGCCTAACCA-3′

mtDNA was amplified with a CFX96 Touch real-time PCR detection system device (Bio-Rad, USA). Each PCR reaction contained 0.4 μL of Encyclo polymerase, 2 μL of 10X Encyclo buffer, 0.2 mM of each dNTP (Evrogen, Russia), 1X SYBR GreenMasterMix (BioDye, Russia), and 0.5 μL of forward and reverse primers in a total volume of 20 μL. qPCR: initial denaturation at 95°С for 3 min; 35 cycles: denaturation 95°С for 30 s, primer annealing at 59°С for 30 s, and elongation at 72°С for 4 min 30 s; final elongation at 72°С for 4 min. The amount of mtDNA damage was calculated per 10000 bp according to the following formula (nulla):

$$\text{Lesions}=\left(1-2^{\left(\Delta \text{long}-\Delta \text{short}\right)}\right)*10000\text{bp}/\text{fragment length}\left(\text{bp}\right),$$

where

Δ long = cq _control_ – cq_experiment_ for 1903-bp fragment

Δ short = cq _control_ – cq_experiment_ for 67-bp fragment

### 2.5 mtDNA copy quantification

mtDNA copy number was determined by qPCR by amplification of mtDNA and genomic DNA (*GAPDH* as a reference gene) with a CFX96 Touch real-time PCR detection system device using qPCRmix-HS SYBR kit (Evrogen, Russia). qPCR cycling conditions were: initial denaturation at 95°С for 3 min followed by 35 cycles: denaturation 95°С for 10 s, primer annealing at 59°С for 30 s, and elongation at 72°С for 1 min. The primers for the *GAPDH* gene (reference) amplification were:

F: 5′-GGCTCCCTAGGCCCCTCCTG-3 ';
R: 5′-TCCCAACTCGGCCCCCAACA-3 '.

The primers used for the mtDNA amplification were:

F: 5′-GGCCACAGCACTTAAACACA-3′
R: 5′-CCCTAACACCAGCCTAACCA-3′

### 2.6 Statistical analysis

The data were analyzed with the STADIA 8.0 software (STADIA, Russia). Comparison of the control and experimental groups was carried out using the Student's *t*-test. The results are presented as mean ± standard mean error. Spearman's rank correlation coefficient was used to estimate the correlation between the H_2_O_2_ concentration in the EBC and amount of lesions in mtDNA, H_2_O_2_ concentration in the EBC and mtDNA copy number, and amount of mtDNA lesions and mtDNA copy number. For each Spearman's test completed in this study, the confidence values (*p*) were estimated. The differences at *p* < 0.05 were considered statistically significant.

## 3 Results

**Figure 1:**
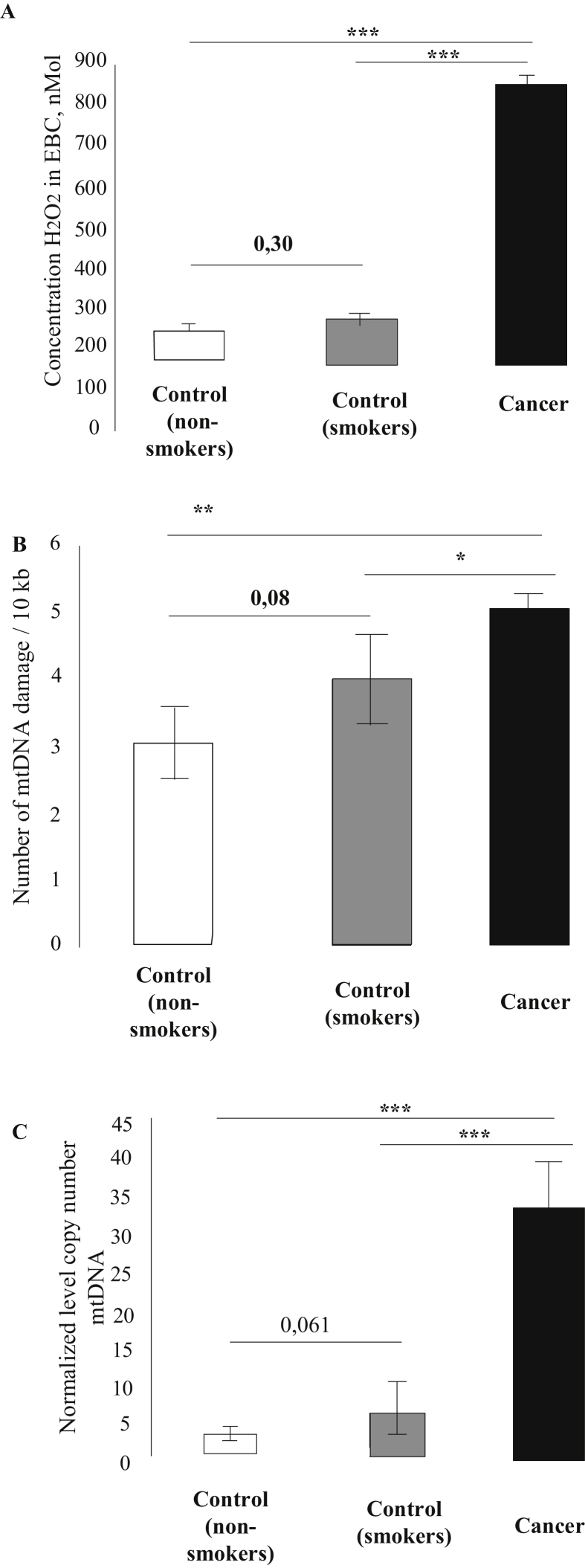
A) H_2_O_2_ concentration in the EBC (mean ± standard mean error) in the studied groups; В) average number of mtDNA lesions in the buccal mucosa cells; С) average mtDNA copy number in the buccal mucosa; ∗∗*p* < 0.01; ∗∗∗*p* < 0.001.

The non-smokers and smokers in the control group demonstrated no difference in the H_2_O_2_ concentration in the EBC (88.3 ± 45.6 nM vs. 128.9 ± 77.6 nM, respectively). The H_2_O_2_ concentration in the EBC of lung cancer patients was 830.1 ± 84.3 nM, which is 9.33 times higher than in healthy non-smokers (*p* < 0.001) and 6.43 times higher than in healthy smokers (*p* < 0.001) (Figure 1A).

The number of DNA lesions in the mtDNA of lung cancer patients was 4.99 ± 0.09 per 10000 bp. The lowest extent of mtDNA damage was observed in healthy non-smokers (2.94 ± 0.58 per 10000 bp; *p* < 0.01) vs. 3.94 ± 0.41 per 10000 bp in healthy smokers (*p* < 0.05). Although smokers demonstrated higher level of mtDNA lesions than non-smokers, the difference between the groups was statistically insignificant (*p* = 0.08) (Figure 1B).

The content of mtDNA (as measured by qPCR in relation to the nuclear DNA) in the buccal cells in the control group was higher in smokers (6 ± 1.8) than in non-smokers (3.06 ± 0.37), but the difference was statistically insignificant (*p* = 0.06). The relative mtDNA content in buccal cells of lung cancer patients was 33.07 ± 6.29, which is 10 times higher than in healthy non-smokers (*p* < 0.001) and 5.5 times higher than in healthy smokers (*p* < 0.001) (Figure 1C).

### 3.1 Correlation analysis

**Figure 2:**
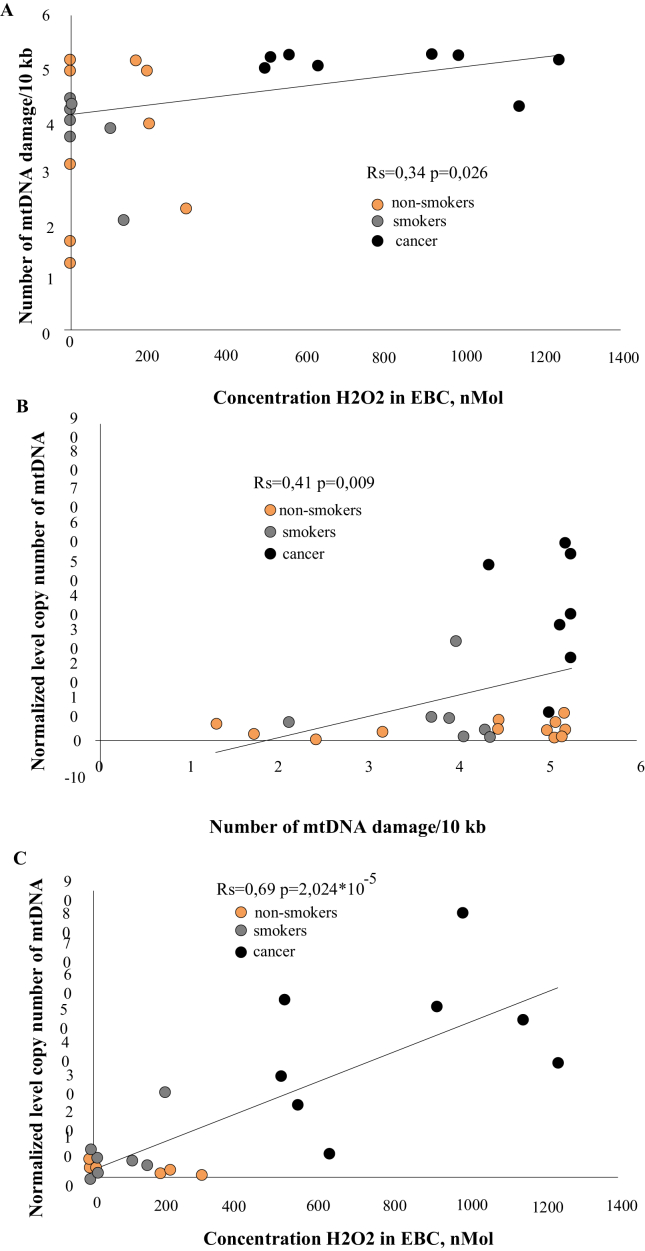
Analysis of correlation between A) H_2_O_2_ concentration in the EBC and amount of mtDNA lesions; В) amount of mtDNA lesions and mtDNA copy number; С) H_2_O_2_ concentration in the EBC and mtDNA copy number. Orange circles, non-smokers; grey circles, smokers; black circles, lung cancer patients.

The correlation between the H_2_O_2_ concentration in the EBC and the number of mtDNA lesions in buccal mucosa cells is shown in Figure 2A; the Spearman's rank correlation coefficient was 0.34 (*p* < 0.05). Figure 2B shows the correlation between the number of mtDNA lesions and mtDNA content in the buccal mucosa cells; the Spearman's coefficient was 0.41 (*p* < 0.01). The correlation between the H_2_O_2_ concentration in the EBC and amount of mtDNA in the buccal mucosa cells is shown in Figure 2C; the Spearman's coefficient is 0.69 (*p* < 0.001).

## 4 Discussion

EBC is used in the diagnostics of various diseases, including lung cancer. EBC contains more than 200 volatile and nonvolatile compounds, whose qualitive and quantitative characteristics can reflect the respiratory tract damage, inflammation-related changes, and effects of therapy (Kuban and Foret, 2013). Carcinogenesis is strongly associated with the action of ROS, H_2_O_2_ being one of the most studied among them (Vilema-Enríquez et al., 2016). In this work, we showed that the H_2_O_2_ concentration in the EBC of lung cancer patients increases compared to the H_2_O_2_ concentration in the EBC of healthy smokers and non-smokers (Figure 1A). These results are in a good agreement with the previously obtained data that the non-small cell lung cancer patients exhale 3.8 times more H_2_O_2_ than healthy subjects (Krawczyk et al., 2017). Tumorigenesis can be accompanied by spontaneous overproduction of ROS by the tumor tissue, which causes imbalance between the levels of oxidants and antioxidants, resulting in oxidative stress (Chan et al., 2009). ROS (including H_2_O_2_) produced by the tumor, are essential for the metabolic adaptation of tumor cells, thus promoting their survival via initiation of redox signaling cascades (Behrend et al., 2003; Han and Chen, 2013). H_2_O_2_ plays a key role in ensuring tumor cell proliferation by inhibiting the antioxidant system (Weinberg and Chandel, 2009). Beside spontaneous ROS generation by the tumor cells, cytotoxic compounds can be also produced by macrophages (Weinberg and Chandel, 2009).

Increased ROS production results in the mutagenic and cytotoxic damage of nuclear DNA which may cause genetic instability and lead to the development of various pathological processes including carcinogenesis (Dizdaroglu, 2015; Levine et al., 2017). mtDNA damage has a stronger effect on ROS metabolism than nuclear DNA damage (Vilema-Enríquez et al., 2016). Our study showed higher number of mtDNA lesions in the buccal mucosa cells of lung cancer patients compared to the control group (Figure 1B). These results are consistent with the data of Yang Ai et al. (2013), who demonstrated that mtDNA mutations are more common in the EBC of lung cancer patients. In many cases, the emergence of mtDNA mutations is associated with the mtDNA damage by ROS (including H_2_O_2_) (Kowaltowski et al., 2009). Therefore, an increased extent of mtDNA damage is a prerequisite for the occurrence of mtDNA mutations.

Increased H_2_O_2_ generation not only increases the extent of mtDNA damage but also leads to the changes in the mtDNA copy number (Piantadosi and Suliman, 2012). We found that the amount of mtDNA in the buccal mucosa cells of lung cancer patients was higher than in the control group (Figure 1C). We also observed the correlation between the H_2_O_2_ concentration in the EBC and mtDNA content. The increase in the mtDNA copy number may be caused by the impairments in the coordinated regulation of mitophagy and mitochondrial biogenesis. Increased H_2_O_2_ production is one of the factors affecting Keap1 oxidation and activation of the Nrf2/ARE signaling cascade (Erlank et al., 2011). This signaling pathway regulates a broad range of mitochondrial functions, including ROS metabolism and mitochondrial biogenesis (Ryoo and Kwak, 2018). Nrf2 regulates expression of nuclear respiratory factor 1 (NRF1), which, in turn, controls expression of TFAM (transcription factor A, mitochondrial). TFAM ensures interaction between mtDNA and DNA polymerase, which leads to the increase in the mtDNA copy number (Valero, 2014). However, the increase in the mtDNA copy number in lung cancer patients was accompanied by the increase in the number of lesions in mtDNA. This might be related to the fact that the damaged mitochondria were not eliminated because of the impairments in mitophagy. Mitophagy, as a special case of autophagy, plays an important role in the maintenance of mitochondrial integrity, which determines the efficiency of respiration and metabolism, thereby modulating tumor growth and metastasis (Vyas et al., 2016). Mitophagy impairments result in the accumulation of defective mitochondria producing ROS (Sena and Chandel, 2012). This causes an instability of the mitochondrial genome and leads to the development of various diseases including lung cancer (Rao et al., 2014). Activation of the Nrf2 cascade promotes cellular antioxidant defense, which should reduce the amount of mtDNA (Lin et al., 2008; Wang and Dai, 2011). However, we observed an increase in the mtDNA content, which contradicts the results of earlier research. Most studies have shown that the mtDNA content in tumor cells might be higher or lower than in normal cells. It remains unclear whether changes in the mtDNA content promote oncogenesis or represent a consequence of carcinogenesis (Yu, 2011; Penta et al., 2001). This issue requires further study.

Earlier, it was shown using the micronucleus test and comet assay that the DNA damage in the buccal mucosa cells is higher in smokers (Di et al., 2019; Gopal and Padma, 2018, Glei et al., 2005). However, we revealed no statistically significant difference in the extent of mtDNA damage in smokers and non-smokers (Figure 1B), which might be related to the fact that no difference in the H_2_O_2_ concentration in the EBC was observed between these groups (Figure 1A). However, we found a trend (*p* = 0.06) for the increased mtDNA copy number in smokers vs. non-smokers (Figure 1C), which is consistent with the results of Tan et al. (2008), who demonstrated an increase in the mtDNA copy number in smokers compared to non-smokers.

We also analyzed whether there is a relationship between the stage and form of cancer and the H_2_O_2_ concentration in the EBC, extent of mtDNA damage, and mtDNA copy number. No difference between these parameters was found (Table 2): the patient with the stage I lung cancer had the same high level of Н_2_О_2_ in the EBC and the amount of mtDNA lesions as patients at stages II, III, and IV. This suggests that the Н_2_О_2_ overproduction in the EBC and increase in the extent mtDNA damage occur at the early stages of lung cancer and, therefore, can be used as markers in the early diagnostics of this disease.

**Table 2:** H_2_O_2_ concentration in the EBC, amount of mtDNA lesions, and mtDNA copy number by group.

	H_2_O_2_ concentration	mtDNA damage, lesions per 10000 bp	mtDNA amount
**Сancer histology**			

**Adenocarcinoma**	81.33 ± 14.50	4.85 ± 0.13	23.26 ± 7.87
**Solid tumor (uncharacterized histology)**	87.85 ± 15.20	4.48 ± 0.66	38.86 ± 7.62
**Other large cell carcinoma, squamous cell carcinoma)**	73.60 ± 18.40	5.25 ± 0.12	52.95 ± 30.1

**Stage**			

**I**	92 ± 0	5.25 ± 0	54 ± 0
**II**	92 ± 0	5.25 ± 0	83.05 ± 0
**IIIA**	96.58 ± 16.17	4,81 ± 0,18	31.28 ± 10.25
**IIIB**	83.11 ± 18.48	4.76 ± 0,66	29.48 ± 7.42
**IV**	63.5 ± 0	5.14 ± 0	8.15 ± 0

**Primary tumor**			

**Tx**	55.20 ± 0	5.25 ± 0	5.25 ± 0
**T1**	N/A	N/A	N/A
**T2**	83.00 ± 6.74	4.60 ± 0.4	38.08 ± 12.6
**T3**	N/A	N/A	N/A
**T4**	96.33 ± 12.79	5.14 ± 0.07	34.62 ± 7.97

**Regional lymph nodes**			

**Nx**	124.50 ± 0	5.23 ± 0	35.48 ± 0
**N0**	72.45 ± 12.47	5.01 ± 0.19	36.34 ± 12.24
**N1**	92.00 ± 0	5.09 ± 0.08	33.02 ± 16.72
**N2**	88.95 ± 25.45	4.72 ± 0.2	23.46 ± 13.24
**N3**	69.85 ± 14.65	3.55 ± 1.71	39.86 ± 17.01

**Distant metastases**			

**Mx**	124.50 ± 0	5.23 ± 0	35.48 ± 0
**M0**	77.18 ± 7.31	4.72 ± 0.3	34.95 ± 7.03
**M1**	63.50 ± 0	5.02 ± 0	8.15 ± 0

We conclude that spontaneous overproduction of ROS (in particular, H_2_O_2_) by the tumor tissue can lead to the changes in the mtDNA copy number, presumably due to the activation of mitochondrial biogenesis and impaired mitophagy. This might lead to the accumulation of defective mitochondria and promotion of mtDNA oxidative damage. Therefore, Н_2_О_2_ overproduction in the EBC, extent of mtDNA damage, and mtDNA copy number can be reliable markers in the early diagnostics of lung cancer.

## Declarations

### Author contribution statement

N.A. Kolbasina: Performed the experiments; Analyzed and interpreted the data; Wrote the paper.

A. Gureev: Conceived and designed the experiments; Analyzed and interpreted the data.

O. Serzhantova, A. Mikhailov and I. Moshurov: Contributed reagents, materials, analysis tools or data.

A. Starkov: Conceived and designed the experiments.

V. Popov: Conceived and designed the experiments; Contributed reagents, materials, analysis tools or data.

### Funding statement

This work was supported by the 10.13039/501100002261Russian Foundation for Basic Research (project 17-29-06036 ofi_m).

### Competing interest statement

The authors declare no conflict of interest.

### Additional information

No additional information is available for this paper.
