# Supplementary material for: Lung cancer increases H2O2 concentration in the exhaled breath condensate, extent of mtDNA damage, and mtDNA copy number in buccal mucosa
Source: Heliyon. 2020 Jun 28;6(6):e04303. doi: 10.1016/j.heliyon.2020.e04303 (PMC7327746; doi:10.1016/j.heliyon.2020.e04303)
Supplement: Supplementary Material [file mmc1.pdf]

Dear Editor of Heliyon!

The team of authors of the article HELIYON-D-19-00314 “Lung cancer increases H_2O_2 concentration in exhaled breath condensate, amount of damage and number of mtDNA copies in buccal mucosa” confirms that they agree with the following order of authors in the final version of the article.

<u>Order of authors:</u>

1) Natalia A. Lutsenko
Department of genetic, cytology and bioengineering, Voronezh State University

2) Artem P. Gureev
Department of genetic, cytology and bioengineering, Voronezh State University

3) Olga V. Serzhantova
Voronezh Regional Clinical Oncological Dispensary
Department of genetic, cytology and bioengineering, Voronezh State University

4) Andrey A. Mikhailov
Voronezh Regional Clinical Oncological Dispensary
Department of genetic, cytology and bioengineering, Voronezh State University

5) Ivan P. Moshurov
Voronezh Regional Clinical Oncological Dispensary

6) Anatoly A. Starkov
Feil Family Brain and Mind Research Institute, Weill Cornell Medicine, New York

7) Vasily N. Popov
Voronezh State University of Engineering Technologies
Department of genetic, cytology and bioengineering, Voronezh State University

<u>Removed author:</u>

Olga Pegina
Voronezh Regional Clinical Oncological Dispensary

Sincerely, author team!
